# Rapid eye movements during sleep in mice: High trait-like stability qualifies rapid eye movement density for characterization of phenotypic variation in sleep patterns of rodents

**DOI:** 10.1186/1471-2202-12-110

**Published:** 2011-11-02

**Authors:** Stephany Fulda, Christoph PN Romanowski, Andreas Becker, Thomas C Wetter, Mayumi Kimura, Thomas Fenzel

**Affiliations:** 1 Max Planck Institute of Psychiatry, Kraepelinstrasse 2, 80804 Munich, Germany; 2 Ingenium Pharmaceuticals GmbH, Fraunhoferstrasse 13, 82152 Planegg, Germany; 3 Psychiatrische Universitätsklinik Zürich, Lenggstrasse 31, 8032 Zürich, Switzerland; 4 Department of Pharmacology and Toxicology, Leopold-Franzens-University, Peter-Mayr-Str. 1, 6020 Innsbruck, Austria

## Abstract

**Background:**

In humans, rapid eye movements (REM) density during REM sleep plays a prominent role in psychiatric diseases. Especially in depression, an increased REM density is a vulnerability marker for depression. In clinical practice and research measurement of REM density is highly standardized. In basic animal research, almost no tools are available to obtain and systematically evaluate eye movement data, although, this would create increased comparability between human and animal sleep studies.

**Methods:**

We obtained standardized electroencephalographic (EEG), electromyographic (EMG) and electrooculographic (EOG) signals from freely behaving mice. EOG electrodes were bilaterally and chronically implanted with placement of the electrodes directly between the musculus rectus superior and musculus rectus lateralis. After recovery, EEG, EMG and EOG signals were obtained for four days. Subsequent to the implantation process, we developed and validated an Eye Movement scoring in Mice Algorithm (EMMA) to detect REM as singularities of the EOG signal, based on wavelet methodology.

**Results:**

The distribution of wakefulness, non-REM (NREM) sleep and rapid eye movement (REM) sleep was typical of nocturnal rodents with small amounts of wakefulness and large amounts of NREM sleep during the light period and reversed proportions during the dark period. REM sleep was distributed correspondingly. REM density was significantly higher during REM sleep than NREM sleep. REM bursts were detected more often at the end of the dark period than the beginning of the light period. During REM sleep REM density showed an ultradian course, and during NREM sleep REM density peaked at the beginning of the dark period. Concerning individual eye movements, REM duration was longer and amplitude was lower during REM sleep than NREM sleep. The majority of single REM and REM bursts were associated with micro-arousals during NREM sleep, but not during REM sleep.

**Conclusions:**

Sleep-stage specific distributions of REM in mice correspond to human REM density during sleep. REM density, now also assessable in animal models through our approach, is increased in humans after acute stress, during PTSD and in depression. This relationship can now be exploited to match animal models more closely to clinical situations, especially in animal models of depression.

## Background

Already in 1875 electrical activity from the brains of rabbits and monkeys was described [1] and by the early 1930s, all major findings in electroencephalograms (EEG) alterations during wakefulness and sleep in general had been discovered [2-6]. It was not until 1953 that rapid eye movement (REM) sleep was first described in humans with rapid, jerky and binocularly symmetrical eye movements as a level of neuronal activity encountered normally during sleep [7]. Since then, these for REM sleep eponymous activities have been found in many non-mammalian (e.g. in birds [8] and reptiles [9]) and mammalian species (e.g. in cats [10-15], rats [16-21] and mice [22-26]).

In humans, REM density - the frequency of REM during REM sleep, increases over the night for successive REM sleep episodes [27-29]. REM density decreases during recovery sleep, after sleep deprivation [30-36] and increases during extended sleep [37-40], with direct relationship to prior sleep duration [33,36]. The occurrence of REM during REM sleep closely relates to the occurrence of ponto-geniculo-occipital (PGO) waves in animals [41] and possibly also in humans [42-44]. In animals these PGO waves have important functions in brain development and plasticity [45]. In humans increased REM density has been found after learning tasks [46-48] and, interestingly, donezepil, an acetylcholinesterase inhibitor that is used in the treatment of Alzheimer's disease, was found to increase REM density and memory in healthy subjects [49-51].

An increased REM density has been found in patients suffering from psychiatric diseases such as post traumatic stress disorder (PTSD) [52] or depression [53]. Indeed, increased REM density has been suggested to be an endophenotype for depression [54,55], as it is systematically elevated in depressed patients [56,57]. REM density has also been associated with treatment outcome [58-60], and in studies of high risk subjects, such as relatives of depressed patients, increases in REM density were present before the onset of the disorder and predicted its development [61-64].

REM sleep and REM density in humans are significantly affected by psychopharmacological drugs. In particular, many antidepressants such as monoamine oxidase inhibitors (MAOI), tricyclic antidepressants and also selective serotonin reuptake inhibitors (SSRI) strongly, rapidly and lastingly reduce REM sleep [65,66]. While these antidepressants suppress the absolute and relative REM sleep amount, REM density increases with continued treatment [67].

It follows that REM density, i.e. the quantitative analysis of REM during REM sleep, is an important parameter in the assessment of human sleep and it would, therefore, be of considerable interest to study this parameter in animals as well.

Standard procedures for obtaining meaningful data on sleep/wake behavior of laboratory animals generally include recordings of EEG and electromyograms (EMG). The techniques of chronic EEG and EMG electrode implantations are rather standardized and routinely utilized in many laboratories. It is remarkable that the recording of REM during sleep has been applied only in very few experiments in basic animal research so far, although, already more than 30 years ago electrooculograms (EOG) were used to characterize basic sleep/wake behavior in mice [22]. In a few, more recent experiments qualitative analyses of EOG were applied to characterize the sleep architecture of mice with a modified serotonin system [26,68] or for the description of ultradian cycles in the same animals [69]. EOG were also applied to characterize sleep/wake behavior of mice treated with hypnotic compounds, used for the treatment of insomnia [70], or to describe the EEG of mice treated with X-rays [71].

To our knowledge none of the previous studies that recorded EOG in rodents presented quantitative analyses of EOG, i.e. assessed REM density. The potential role of such an approach lies in an in-depth characterization of sleep/wake behavior in animal models, for example for depression, which could then be more closely related to reports from human studies, where REM density in REM sleep is a standard parameter in data collection and data interpretation. It would also have the potential to characterize and evaluate the effect of medications with a closer relation to clinical situations.

In the present study, we introduce a standardized and well-defined approach to measure and automatically analyze REM during sleep in mice.

## Methods

### Animal housing

6 adult mice (C57BL/6J, Harlan-Winkelmann, Germany) were used in the experiments. All animals were housed in individual recording cages located in a sound attenuated chamber at constant temperature (22 ± 1°C) and controlled light-dark cycles (12-12 h, lights on: 12 am at 100l×) during habituation, recovery from surgery and during all recording sessions. Food and water were available *ad libitum*.

### Experimental design

All animals were adapted to the light-dark cycle for seven days before surgery. After surgery all animals were allowed to recover for another 14 days before two days of baseline recordings started (B1; B2), followed by two days of treatment recordings (T1; T2). During treatment, each animal received two oak flakes per day, saturated with the following solution: 0.2% saccharine, 0.05% vanillin in 5 μl aqua dest./oak flake. To ensure acceptance of the oak flakes at ZT0, all animals where food deprived for four hours prior to its presentation. This treatment was designed in anticipation of future studies, where we plan for the application of several antidepressants via this routine to systematically study the effects of these drugs on REM during sleep. After termination of the experiments animals were euthanized with an overdose of sodium pentobarbital.

### Surgery

**Figure 1:**
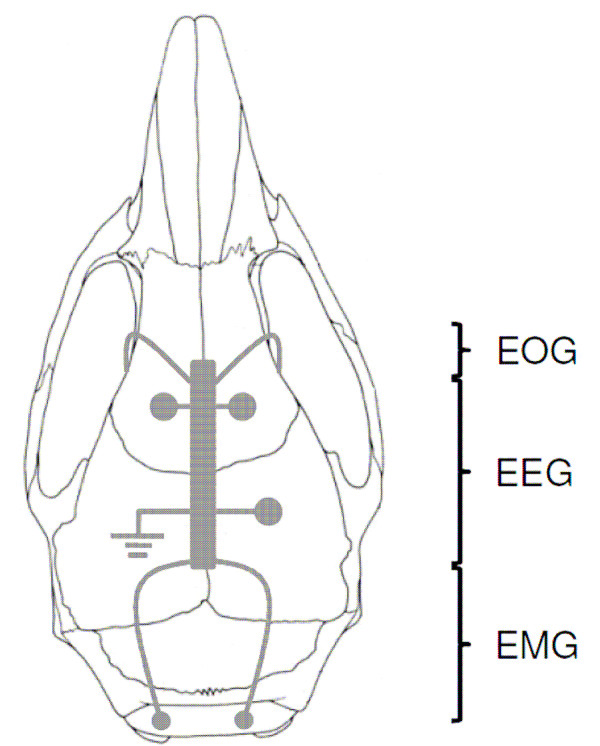
**Dorsal view of the skull illustrating the placement of electrodes**. The EOG electrodes penetrated the connective tissue separating the orbit from the frontal bone (*os frontale*) bilaterally. Both electrodes were gently born around the *musculus rectus lateralis *without penetrating the muscle tissue. The two rostral EEG electrodes were placed centrally at the frontal bone, a third EEG electrode and one grounding electrode were placed caudally at the parietal bone (*os parietale*). Two EMG electrodes were inserted in neck muscles bilaterally (adapted from: www.informatics.jax.org).

During surgery the animals were fixed in a stereotaxic apparatus. For anaesthesia a isofloran/oxygen mixture was constantly delivered through a respiratory mask (custom made) and body temperature was maintained with a small heating pad. Before and after surgery, all animals received atropine sulphate (0.05 mg/kg BW, Braun Melsungen, Germany) and meloxicam (0.5 mg/kg BW, Braun Melsungen, Germany). Two EOG electrodes were placed bilaterally on the dorsal field of the eye muscles within the orbit (please refer to Figure 1 for details). These electrodes where insulated with cured coating (Konform SR, ITW Chemitronics, United Kingdom) prior to implantation, only the tip of the electrodes had ohmic contact with the surface of the eye muscles. Four EEG electrodes where placed epidurally on the intact cerebral membranes of the cortex through small holes in the skull (diameter: ca. 250 μm). Two electrodes were inserted into the neck muscle to record EMG signals. All electrodes (gold wire, diameter: 150 μm, with ball-shaped endings) were soldered to a connector (PCB socket connector) which was affixed to the skull with Paladur and two jeweller's screws. The position of each individual recording electrode and especially the EOG electrodes were carefully inspected for proper placement and possible inflammations at tissue surrounding the electrode tips after termination of the experiment. Only animals without any tissue abnormalities and proper placement of the EOG electrodes were used for further analyses.

### EOG/EEG/EMG recordings

After surgery, animals were connected to an 8-pole recording cable. EEG, EMG, and EOG signals were relayed via the sliding contact of a head unit that was attached to a counter-balanced swivel system, and which allowed for free and undisturbed movement of the animals during recordings. All signals were fed into a preamplifier (1000×, custom made) and main amplifier (10×, custom made). The EEG and EOG signals were analog band-pass filtered (0.5-29 Hz, filter frequency roll off 48 dB/octave) and analog-to-digital converted at a sampling rate of 64 Hz (AD board, NI PCI-6070, National Instruments, Austin, USA). Root mean square was applied to the non-filtered EMG signals before its digital conversion (sample rate: 64 Hz). The digital data were stored for further offline-analysis.

### Automated scoring of eye movements

The Eye Movement scoring in Mice Algorithm (EMMA) conceptualized REM as singularities of the EOG signal and used wavelet methodology to detect them [72]. The EOG signal was wavelet filtered, eliminating the lower frequencies, and a continuous wavelet transform with the Mexican hat wavelet was computed. Wavelet transform modulus maxima were used to identify singularity candidates at the smallest scale against a threshold derived from the variation of the wavelet transform modulus. These candidates were subjected to two thresholds: a) an amplitude criterion (> 4*85^th ^percentile of the signal or < 4*15^th ^percentile of the signal) to ensure that REM was decidedly higher than the baseline signal and to protect against regular high frequency artifacts; b) the absolute lag-2 difference of the signal had to be higher than 2*95^th ^quantile of the lag-2 differences of the signal that did not include REM candidates. All threshold criteria were derived from a training set that was independent from the validation set (see below). Automated detection was programmed in R [73] and made use of the Rwave package [74]. Details of the EMMA algorithms are given in the appendix.

Each REM was conceptualized as having a start, a peak, and a stop and each of these time points was defined in terms of peaks of the signal. Peaks were defined as points where the direction of the signal changed from downward to upward deflection or vice versa. The peak of REM was detected with the EMMA algorithm, the start was defined as the nearest change point before the peak and the end was the nearest change point after the peak. A complete REM, therefore, consisted of a down- or upward initial deflection and the following opposite deflection.

The following parameters were extracted for each REM: amplitude (measured in mV at the peak of REM), duration of REM (measured from start to end), velocity of the initial and opposite deflection (measured in mV/ms for start to peak and peak to stop of REM), and direction of REM (upward or downward deflection).

### Validation of automated scoring of REM

The automated scoring was validated against visual scoring by two of the authors (SF, TF) in 260 randomly selected epochs (130 REM sleep, 130 non rapid eye movement sleep (NREM sleep)) recorded at B1. A comparison between the two visual scorers showed a sensitivity of 92% with a specificity of 86% yielding a positive predictive value (PPV) of 79% and a negative predictive value (NPV) of 91%. Comparison of automated scoring and visual scoring showed comparable indices with a sensitivity of 84% and a specificity of 87% (PPV 81%; NPV 89%) for the first scorer and 86% for both sensitivity and specificity for the second scorer (PPV 79%, NPV 91%).

### Data analysis

The vigilance states WAKE, NREM sleep and REM sleep were scored semi-automatically using a FFT-algorithm spectral analysis from all recordings on a LabVIEW^®^-based scoring program (SEA, Koeln, Germany). The frequency bands were as follows: δ (0.75-5 Hz), θ (6-9 Hz), α (10-15 Hz), η (16-22.75 Hz) and β (23-31.75 Hz). A detailed description of the scoring procedure is described elsewhere [75,76]. All semi automatically scored vigilance states were inspected manually and rescored if needed.

All three vigilance states (WAKE, NREM sleep and REM sleep) were calculated in consecutive two hour means. Effects of treatment and time intervals were evaluated for 23 hours without a separation in dark and light period with 2-factorial analyses of variance with a repeated measures design. Significant main or interaction effects in ANOVA were followed by a post hoc test for simple effects (Student-Newman-Keuls test, α = 0.05).

REM density and REM parameters were computed separately for each animal and day, for REM sleep and NREM sleep and for different time spans (whole day, light and dark period, 2 hour intervals). Comparison between sleep stages, days and time periods were undertaken with linear mixed models [77] that treated animals as the random factor. In addition, we quantified trait-like stability of REM density and sleep stage duration with the intraclass correlation coefficient (ICC). The ICC was computed as the ratio of between-animal variance to total variance with variance derived from a linear mixed model analysis. ICC values were interpreted according to benchmark ranges as "slight" (0-0.2), "fair" (0.2-0.4), "moderate" (0.4-0.6), "substantial" (0.6-0.8) and "almost perfect" (0.8-1.0) [78]. Data analysis was undertaken with R [73] and the nlme package [79] in R.

### Animal care

Laboratory animal care and experiments were conducted in accordance with the regulations of the current version of German Law and Animal Protection. Animal protocols were approved by the Government of Bavaria.

## Results

### A) General sleep/wake behavior

**Figure 2:**
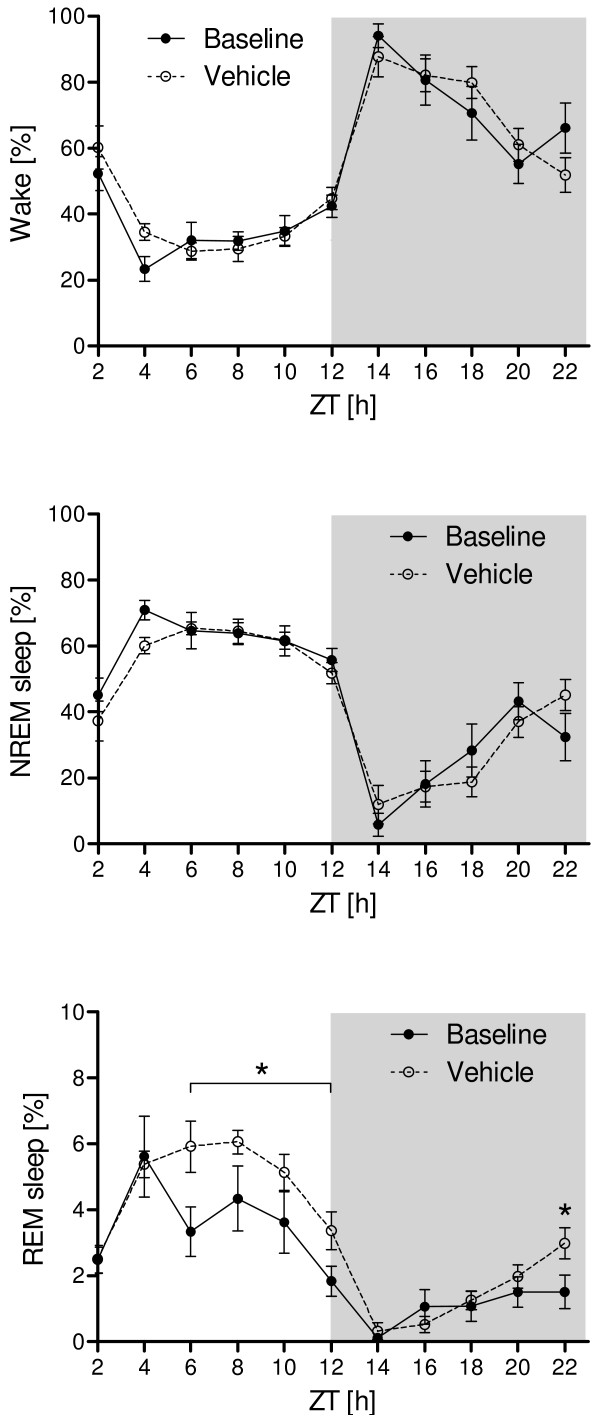
**Distribution of vigilance states**. Typically for a nocturnal rodent, wakefulness was strongly reduced during the light period (ZT0-ZT12), compared to the dark period (ZT12-ZT22). Inversed patterns were obtained for NREM sleep and REM sleep with significant differences between baseline and treatment recordings only for REM sleep (asterisks). Data points represent two hour means ± SEM. Gray background indicates the dark period.

The analysis of the vigilance states revealed small amounts of wakefulness and large amounts of NREM sleep during the light period and increased wakefulness and decreased NREM sleep during the dark period (Figure 2). For the vigilance states WAKE (F_(5,11) _= 1.805; p_time vs. treatment _= 0.075) and NREM sleep (F_(5,11) _= 1.513; p_time vs. treatment _= 0.154) no statistically significant interactions were found. As for NREM sleep, the amount of REM sleep was highest during the light period and decreased to its nadir towards the first two hours of the dark period. Significant differences in REM sleep between baseline and treatment were found at ZT6- ZT12 and ZT22 (F_(5,11) = _2.705; p_ZT6 _< 0.001; p_ZT8 _= 0.019; p_ZT10 _= 0.035; p_ZT12 _= 0.035; p_ZT22 _= 0.042).

### B) REM characteristics

#### Proportion of REM in REM bursts

**Figure 3:**
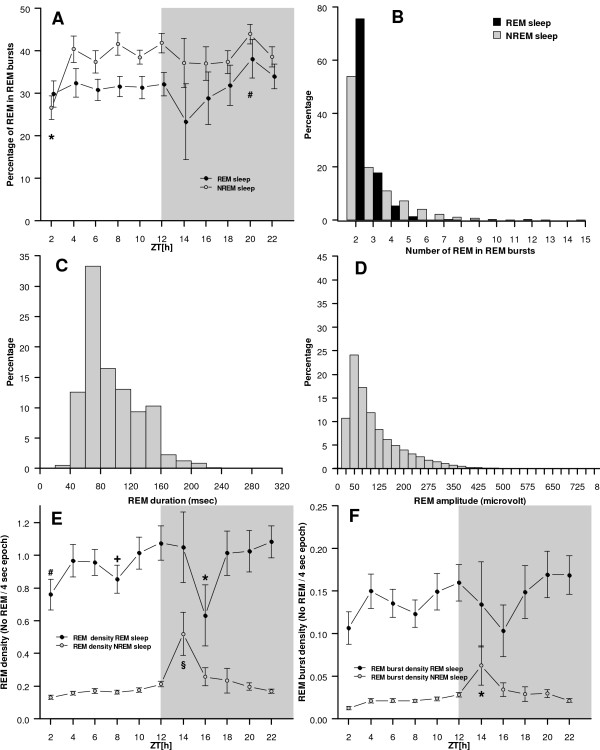
**Description of REM**. **A**: Proportion of REM in REM bursts differed across the recording period. In addition, the proportion was lower during REM sleep than NREM sleep (* The proportion was lower at ZT2 than at ZT8-ZT12 and ZT18-ZT22 (p < 0.05); **# **The proportion was higher at ZT20 than at ZT5 and ZT13 (p < 0.05)). **B**: Number of REM in REM bursts. REM bursts during NREM sleep tended to include a higher number of REM than during REM sleep. **C**: Distribution of all recorded REM durations. REM duration was computed as the difference in milliseconds between the start and the end of the REM. **D**: Distribution of all recorded REM amplitudes. REM amplitude was computed as absolute difference from zero at the peak of the REM in mV. **E**: REM density in REM sleep and NREM sleep differed across the recording period. REM density during NREM sleep showed increased density at the beginning of the dark period. (* REM sleep REM density at ZT16 was significantly lower than ZT4 to ZT6; ZT10 to ZT14, and ZT18 to ZT22; **# **REM sleep REM density was significantly lower at ZT2 than ZT10, ZT12, and ZT18 to ZT22; **+ **REM sleep REM density was significantly lower at ZT8 than ZT12 and ZT22; **§ **NREM sleep REM density was significantly higher at ZT14 than all other time intervals; (p < 0.05 for all comparisons)). **F**: REM burst density in REM sleep did not differ across the light and dark period. REM density during NREM sleep showed increased density at the beginning of the dark period (ZT14-ZT22). A REM burst was defined as at least two eye movements not faster than 200 ms apart (* NREM sleep REM burst density at ZT14 was significantly higher than at all other times (p < 0.05)).

For all animals and days, 37% of all REM occurred in bursts of at least two individual eye movements. This percentage was higher for NREM sleep (41%) than REM sleep (33%, F_(1,29) _= 21.16, p < 0.001). There was no overall difference between the light and the dark period but we found a *day × light/dark *interaction (F_(2,61) _= 3.43, p = 0.0387) where only for day B2 the percentage of REM in bursts was lower for the light period than the dark period (34% vs. 41%, p < 0.05) while for the two following recording days (T1, T2) there was no difference. The proportion of REM in bursts differed across 2-hour intervals (F_(10,347) _= 2.25, p = 0.015) and was lower at the beginning of the light period (ZT2) and higher at the end of the dark period (ZT20, p < 0.05) (Figure 3A). In addition, REM bursts during NREM sleep tended to consist of a higher number of REM (Figure 3B).

#### Direction of REM

Overall, as many downward as upward deflections of REM were recorded (54% vs 46%, F_(1,65) _= 1.19, p = 0.2795). The proportion of upward deflections tended to be less for REM sleep than NREM sleep (42% vs 48%, F_(1,29) _= 5.84, p = 0.0221). There was no difference for the proportion of upward deflections between the light and the dark period or between 2-hour time intervals.

#### Duration of REM

The average duration of all REM was 95 ± 36 ms and the distribution of all individual REM durations is given in Figure 3C. REM duration was longer during REM sleep than during NREM sleep (104 ± 4.8 vs. 90 ± 3.5, F_(1,63) _= 167.36, p < = .001). REM duration was also longer for REM in bursts (97 ± 3.5 vs. 92 ± 3.9, F_(1,63) _= 15.92, p < 0.001) but these two factors interacted significantly (sleep stage × burst interaction, F_(1,63) _= 16.26, p < 0.001). Specifically, during REM sleep, REM duration did not differ between isolated REM and REM in bursts (104 ± 4.9 vs. 104 ± 6.0) while during NREM sleep isolated REM were shorter than REM in bursts (87 ± 4.3 vs. 95 ± 3.7, p < 0.05). There was no difference in REM duration between the light and the dark period. REM duration varied across 2-hour intervals (F_(10,698) _= 4.056, p < 0.001) with REM duration being shorter at the beginning of the dark period (ZT14 and ZT16 vs. all other intervals, p < 0.05).

#### Amplitude of REM

The average magnitude of the amplitude of all REM was 124 ± 83 mV (including amplification) and the distribution of all individual REM amplitudes is given in Figure 3D. Similar to REM duration, the amplitude was lower for REM during REM sleep than NREM sleep (F_(1,63) _= 365.82, p < 0.001), it was lower for isolated REM compared to REM in bursts (F_(1,63) _= 533.16, p < 0.001), but there was also a significant interaction between sleep stage and burst status (F_(1,63) _= 261.14, p < 0.001). In particular, although the amplitude was lower during REM sleep than NREM sleep for both isolated and burst REM, the increase in amplitude from isolated to burst REM was significantly larger during NREM sleep (from 96 ± 18.2 to 193 ± 18.0) than during REM sleep (from 89 ± 8.5 to 106.14.9, p < 0.05). REM amplitude did not differ between the light and dark period or across 2-hour intervals.

### C) REM density and REM burst density

REM density was computed as the number of REM per 4s epochs. We computed both total REM density and REM burst density. For the total recording period, REM density was significantly higher during REM sleep than during NREM sleep (0.96 ± 0.33 vs. 0.18 ± 0.06, F_(1,29) _= 203.709, p < 0.001). While there was no systematic difference in REM density between the light and the dark period, for both REM sleep and NREM sleep, REM density differed across the recording period (Figure 3E) with a significant interaction between sleep stage and time interval (F_(10,340) _= 2.009, p = 0.0317). For NREM sleep, REM density peaked at the beginning of the dark period. For REM sleep, REM density showed ultradian changes with troughs at ZT2, ZT8 and ZT16 (Figure 3E).

Density of REM bursts (burst density) was significantly higher for REM sleep than for NREM sleep (0.14 ± 0.06 vs. 0.02 ± 0.01, F_(1,29) _= 121.939, p < 0.001). Again, there was no difference between the light and the dark period. Although burst density during REM sleep varied similar to REM density during REM sleep, there was no significant difference between 2-hour intervals (F_(10,158) _= 1.185, p = 0.305). In contrast, burst density during NREM sleep again peaked at the beginning of the dark period (Figure 3F; F_(10,177) _= 2.975, p = 0.0017).

#### Trait-like stability of REM density

**Figure 4:**
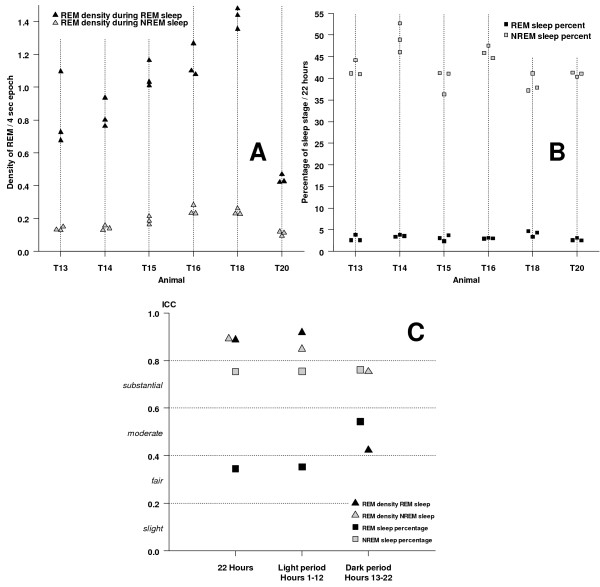
**Trait-like stability of REM density**. **A**: REM density during REM sleep and NREM sleep per animal per night (three different nights are separately plotted). **B**: REM sleep and NREM sleep percentage per animal per night. **C**: Trait-like stability of REM density and sleep stage proportion, quantified as ICC from a linear mixed model analysis (see statistical analysis).

We used ICCs to quantify trait-like stability of REM density during REM sleep and NREM sleep. For descriptive purposes the stability of REM sleep and NREM sleep duration is given as a further comparison standard. Figures 4A and 4B give the individual REM density and sleep stage proportions per animal and night. Trait-like stability, quantified as the ratio of between-animal variance to total variance, was almost perfect for REM densities during REM sleep and NREM sleep for the total recording period or the light period (Figure 4C). For the dark period alone, REM density during REM sleep was only moderate, while REM density during NREM sleep was substantial. As a comparison standard, stability of REM sleep proportion was only fair for the total recording period and the light period and moderate for the dark period. In contrast, stability of NREM sleep proportion was substantial for all three periods. For the total recording period and the light period, trait-like stability was higher for REM densities than sleep stage proportions.

### D) REM bursts and micro arousals

**Figure 5:**
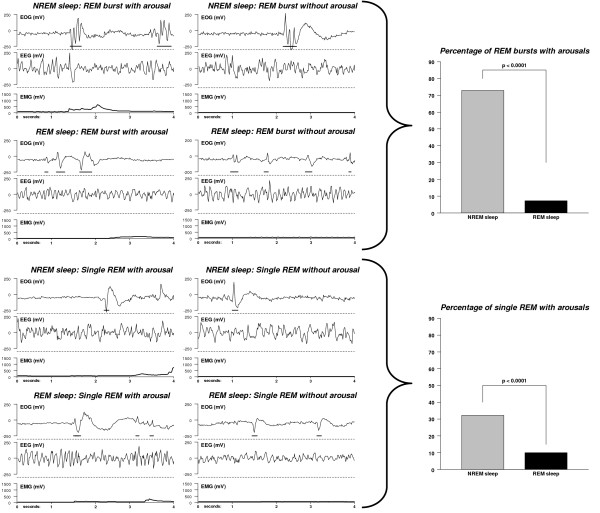
**REM and REM bursts and micro arousal during sleep**. **Left and middle panels**: Examples of REM and REM bursts in REM sleep and NREM sleep associated with micro-arousals or without micro arousals. **Right upper panel**: REM bursts were more frequently associated with micro-arousals during NREM sleep than during REM sleep. **Right lower panel**: Single REM were more frequently associated with micro-arousals during NREM sleep than during REM sleep.

Given that during NREM sleep REM bursts were relatively more frequent than isolated REM, and compared to REM sleep, REM bursts during NREM sleep consisted of more REM with a higher amplitude and a longer duration, as well as the peak of REM bursts at the beginning of the dark period for NREM sleep, we explored whether REM bursts during NREM sleep could be associated with transient micro arousals defined as short EMG increases (< 4 sec). To that end, we randomly chose 360 epochs with REM bursts (10 epochs × 6 animals × 3 days × 2 sleep stages) and 360 epochs with isolated REM. At the time of evaluation we were blind with regard to sleep stage, day or animal. The majority of REM bursts during NREM sleep were associated with micro arousals (72.8%) while this percentage was significantly lower during REM sleep (7.2%; Chi^2 ^= 158.4, df = 1, p < 0.0001, Figure 5). Similarly, single REM were associated more frequently with micro arousals during NREM sleep than during REM sleep, however, in an overall lower proportion (32.2% vs. 10.0%, Chi^2 ^= 25.4, df = 1, p < 0.0001).

## Discussion

Standard procedures to obtain meaningful data about the sleep/wake behavior of laboratory animals generally include recordings of the EEG and the EMG. The techniques of chronic EEG and EMG electrode implantations are routinely utilized in many laboratories. Sophisticated algorithms and the experience of the researchers in the handling of data being obtained in basic research with laboratory animals allow differentiation between wakefulness, NREM sleep and REM sleep. In the present study we provide a complementary element of sleep data analysis, based on a highly standardized evaluation of eye movements (EMMA). We found that REM density recorded across three nights showed remarkable intra-individual trait-like stability. REM density was significantly higher during REM sleep than during NREM sleep and this sleep-stage specific distribution corresponds with human REM density. While REM density during REM sleep showed an ultradian course, during NREM sleep REM density peaked at the beginning of the dark period. The majority of single REM and REM bursts were associated with micro arousals during NREMS, but not during REMS.

The present configuration of the EOG electrodes was designed to record eye muscle activities per se with the electrode placed between the musculus rectus lateralis and musculus rectus superior. The gold wires used in our study could be replaced by multiple wires such as those used to record with tetrodes. Single wires could then be brought individually to the musculus rectus superior and the left and right musculus rectus lateralis. Although not part of our study, this extension of the present electrode configuration would allow differentiating between dorsal and lateral eye movements. Provided that the morphological components of the recorded potentials from separated dorsal and lateral eye movements are similar to the potentials we obtained, the EMMA algorithm could be applied with only minor adaptations in threshold detection. We doubt that the musculus rectus inferior could be equipped solidly with electrodes to record ventral eye movements with the present approach.

We found REM not only during REM sleep but also during NREM sleep in mice. One possible concern could therefore be that REM during NREM sleep are due to the EEG signals, in particular the large slow potentials, contaminating the EOG signal. We judge this as unlikely for several reasons. First, we designed a wavelet filter that eliminated lower frequencies (0.25 to 2 Hz) from the EOG signal before REM detection. Second, EOG electrodes where fully isolated with coating against ohmic contact on the skull's surface, leaving only the non-isolated electrode tip embedded fully in the eye muscle without contact to the bone. Third, detection of REM was based on the concept of singularities i.e. discontinuities in the signal that are not continuous on a particular derivative. To be considered a REM candidate, a local maximum at the finest scale (fastest frequency) of the wavelet transform modulus maxima was a necessary (though not sufficient) condition. Therefore only high frequency noise can trigger false detection. We would expect slow EEG components leaking into the EOG signal to also affect neighboring signal points and our threshold based on the standard deviation of the local wavelet transform modulus maxima was chosen to protect against this possibility.

Given that the EOG electrodes are chronically implanted together with the EEG and EMG electrodes, any experimental recording situation in freely moving animals is conceivable. We applied the present electrode configurations for several weeks in all of our animals and detected neither any behavioral irritations, such as scratching the eye lids, nor inflamed tissue post mortem.

One possible limitation is the low sampling rate of 64 Hz in the present study. We can not exclude the existence of REMs with higher frequencies (> 29 Hz) and therefore our results may be an underestimation of the frequency of REMs during sleep in mice. Future studies with higher sampling rates are needed to clarify this issue.

In the present study, single REM and REM bursts were associated with micro-arousals, which we defined as transient EMG increases during NREMS, but not during REMS. The definition found in the literature for arousals is inconsistent. Single events of arousals within a 4 s epoch or a waking episode lasting less than 16 seconds were defined as sleep fragmentations and separated from clear transitions between vigilance states which were longer than 16 seconds [80]. Other definition criteria for clear changes to another behavioral state comprised eight or more consecutive 4s epochs scored as one behavioral state, which were followed by eight or more consecutive 4s epochs scored as a different behavioral state [81]. In other studies, micro-arousals were defined as events in NREM sleep lasting 5-15s and including a drop of at least 50% in the EEG power in the δ band [82]. Short, single episodes in NREMS with increased power in the θ band and EMG activation were counted as wake [82]. In the present study, transient EMG activity (< 4s) during sleep was taken to define micro arousals. When evaluating micro-arousals we found EMG increases both with and without changes in the EEG. Both types of micro-arousals were frequently accompanied with either single REM or REM bursts (Figure 5 gives examples for both events). In addition to what is defined as a state of arousal in the literature, the present data suggest differentiating micro-arousals in more detail. We would suggest reserving the concept of "micro-arousal" for events less than 8 seconds. Within this time frame, micro arousals could be separated between micro-arousal with EMG changes and micro arousal without EMG changes. Additionally, micro-arousals could be divided into events with single REM and with REM bursts. The occurrence of REM in the majority of micro-arousals in the present study may also support the need to implement the eye movements per se as a general feature in the detection and declaration of micro-arousals. Since arousals are deeply involved in the pathophysiology of human sleep disorders [83], the establishment of well-defined micro-arousals in mice may also help to better understand the origin of increased micro-arousals in particular sleep disorders and to characterize their sleep more fully.

Rapid eye movements, which gave name to the prominent vigilance state of REM sleep in humans, had never really been in the focus of in-depth investigations in animals, although, general references to eye movements during sleep and wakefulness were obtained in several non-mammalian species and mammalian species (please refer to the introduction). This obviously fundamental and phylogenetically diverse component of REM sleep seems to be predestined to serve as a tool in translational approaches between basic and clinical research.

In humans REM density is an important parameter that has been associated with sleep satiety [37], learning and memory [46-48] and psychiatric disorders [52]. Importantly, increased REM density has been proposed to be an endophenotype for depression [54,55] and is sensitive to treatment with antidepressants. Characterization of sleep in mouse models of depression has shown REM sleep facilitation and increased sleep fragmentation that resemble endophenotypes of depressed patients [84-88].

Analysis of REM density is expected to complement and significantly extend sleep characterization in these mouse models. Importantly, we found a high intra-individual stability of REM density across several days with almost perfect trait-like stability that was substantially higher than the stability of REM sleep proportion. REM density might therefore be better suited to characterize phenotypic variations in sleep patterns in mice.

## Conclusions

REM density plays a prominent role in psychiatric diseases. We have developed the Eye Movement scoring in Mice Algorithm (EMMA) to detect REM as singularities of the EOG signal, based on wavelet methodology, and characterized eye movements during sleep in mice. REM density was significantly higher during REM sleep than NREM sleep in mice and across three nights showed a high trait-like stability that outperformed the stability of REM sleep proportion. During NREM but not REM sleep the majority of REM bursts were associated with micro-arousals. Automated detection and analysis of REM density in mice is expected to contribute significantly to the characterization of sleep in mouse models of psychiatric disorders.

## Authors' contributions

TF designed the study, developed the EOG electrodes and implantation procedures, made the experiments, evaluated the data and wrote the manuscript; SF designed the study, developed EMMA, evaluated the data and wrote the manuscript; AB made the experiments; CPNR evaluated the data; TCW and MK critically revised the manuscript. All authors read and approved the final manuscript.

## APPENDIX

### Eye Movement in Mice scoring Algorithm (EMMA)

Eye movements were detected separately for each epoch (4s or 256 sampling points) of sleep. To avoid phenomena associated with the edges of the epoch, the detection algorithm was used on three overlapping windows -1s to 3s, 0 to 4s, and 1 to +1s.

1) The EOG signal was filtered with a low frequency filter that used discrete dyadic wavelet transform with Mallat's wavelet and with 8 decomposition scales. Scales 4 to 8 were set to 0 and the resulting signal was subjected to an inverse dyadic wavelet transform. This procedure omitted frequencies centered at 0.25 to 2 Hz. The choice of Mallat's wavelet and the dyadic transform ensures that the reconstructed signal preserves locations and values at the extrema.

2) Rapid eye movement (REM) candidates were detected as singularities candidates from the wavelet transform modulus maxima of a continuous wavelet transform [72]. We used the complex-valued 2^nd ^derivative of the Gaussian wavelet, the Mexican hat wavelet, with 2 moments on an 8 octave scale with 8 scales within each octave. REM candidates were local maxima at the finest scale of the wavelet transform modulus maxima (WTMM) that were above a threshold based on the standard deviation of the WTMM and the sampling length [(standard deviation of the modulus)*square root (2*log(sample length)) ] [89].

3) Singularity candidates from the WTMM were then compared to two thresholds: first, peak amplitudes of the eye movement candidates had to be clearly above the background noise (> 4*85^th ^percentile or < 4*15^th ^percentile of the signal amplitude). Second, instantaneous amplitude, computed as absolute lag-2 difference, had to be above the 2*95^th ^quantile of the absolute lag-2 differences of the signal, that did not include the eye movement candidates. This latter threshold was used to protect against regular high frequency artifacts.

4) Each REM was conceptualized as having two phases: a significant deflection from baseline and the subsequent return to baseline. In the case of a positive deflection, the start of the REM was the closest local minimum before the singularity, the peak of the REM the closest local maximum after the singularity, and the end of the REM the next local minimum after that. In case of a negative deflection local minima were exchanged for local maxima and vice versa.

5) In the case that two or more singularities were adjacent to each other, the number of different REM were determined according to the first threshold criterion

6) (> 4*85^th ^percentile or < 4*15^th ^percentile of the signal): to detect a second REM subsequent to a first one, where the peak of the first REM (which is above the threshold) was the start of the second REM, the peak of the second REM had to be above the threshold too.
